# Multi-scale characterization of symbiont diversity in the pea aphid complex through metagenomic approaches

**DOI:** 10.1186/s40168-018-0562-9

**Published:** 2018-10-10

**Authors:** Cervin Guyomar, Fabrice Legeai, Emmanuelle Jousselin, Christophe Mougel, Claire Lemaitre, Jean-Christophe Simon

**Affiliations:** 1 INRA, UMR 1349 INRA/Agrocampus Ouest/Université Rennes 1, Institut de Génétique, Environnement et Protection des Plantes (IGEPP), Le Rheu, France; 2 Université Rennes 1, Inria, CNRS, IRISA, F-35000 Rennes, France; 3 INRA, UMR CBGP (INRA/IRD/Cirad/Montpellier SupAgro), Campus International de Baillarguet, Montpellier, France

**Keywords:** Host-microbiota interactions, Aphids, Metagenomics, Symbiosis, Phylogeny

## Abstract

**Background:**

Most metazoans are involved in durable relationships with microbes which can take several forms, from mutualism to parasitism. The advances of NGS technologies and bioinformatics tools have opened opportunities to shed light on the diversity of microbial communities and to give some insights into the functions they perform in a broad array of hosts. The pea aphid is a model system for the study of insect-bacteria symbiosis. It is organized in a complex of biotypes, each adapted to specific host plants. It harbors both an obligatory symbiont supplying key nutrients and several facultative symbionts bringing additional functions to the host, such as protection against biotic and abiotic stresses. However, little is known on how the symbiont genomic diversity is structured at different scales: across host biotypes, among individuals of the same biotype, or within individual aphids, which limits our understanding on how these multi-partner symbioses evolve and interact.

**Results:**

We present a framework well adapted to the study of genomic diversity and evolutionary dynamics of the pea aphid holobiont from metagenomic read sets, based on mapping to reference genomes and whole genome variant calling. Our results revealed that the pea aphid microbiota is dominated by a few heritable bacterial symbionts reported in earlier works, with no discovery of new microbial associates. However, we detected a large and heterogeneous genotypic diversity associated with the different symbionts of the pea aphid. Partitioning analysis showed that this fine resolution diversity is distributed across the three considered scales. Phylogenetic analyses highlighted frequent horizontal transfers of facultative symbionts between host lineages, indicative of flexible associations between the pea aphid and its microbiota. However, the evolutionary dynamics of symbiotic associations strongly varied depending on the symbiont, reflecting different histories and possible constraints. In addition, at the intra-host scale, we showed that different symbiont strains may coexist inside the same aphid host.

**Conclusions:**

We present a methodological framework for the detailed analysis of NGS data from microbial communities of moderate complexity and gave major insights into the extent of diversity in pea aphid-symbiont associations and the range of evolutionary trajectories they could take.

**Electronic supplementary material:**

The online version of this article (10.1186/s40168-018-0562-9) contains supplementary material, which is available to authorized users.

## Background

Symbioses have been studied for long in the case of simple binary interactions between a host and a single symbiont. Many studies have unveiled the functional impacts and the evolutionary consequences of these symbioses including acquisition of novel functions, transmission patterns [1, 2], genomic changes [3], reproductive manipulations (reviewed in [4]), or cost/benefit balance of symbiotic relationships [5, 6]. Yet, the advances of molecular techniques in the last decades have revolutionized the description and understanding of host-microbe interactions and revealed that every plant or animal is interacting in some way with multiple microbes [7]. Biology is undergoing a paradigm shift where individual phenotypes should be considered as resulting from the combined expression of the host and associated microbe genomes (metagenomes) [8]. As a reflection of this conceptual shift, the term “holobiont” is now used to name the complex ecosystem of a host and its community of associated organisms [9, 10]. Similarly, the term “hologenome” is used to describe the collection of genomes of a host and its microbiota [11]. A prerequisite to understand the functional, ecological, and evolutionary implications of host-microbiota associations for holobionts is to evaluate the extent and partitioning of diversity at different scales involving individuals and populations of holobionts. This can be obtained from (i) a full inventory of the microbial entities associated with the host, including transient low abundant symbionts and (ii) a fine characterization of the genomic diversity of microbial partners both within and between individual hosts from different populations. Inter-individual host diversity is often ignored when pooling together several individuals, or underestimated by insufficient sampling in the population, and intra-host variability is rarely considered, but these two levels are essential to infer the evolutionary dynamics of host microbiota interactions [12] and to better link microbiota diversity with associated phenotypic changes in the host [13].

Next generation sequencing techniques can provide whole genome sequencing data of communities of organisms. Some host sequencing projects contain microbe-related reads that are often considered as “contaminant” in the analysis of the host genome. These datasets can actually be analyzed and provide meaningful insights about organisms seen as holobionts. Shotgun metagenomic sequencing has several features which enables high-resolution analysis of taxonomic and genetic diversity associated with holobionts. First, because it is a without a priori technique, it can capture all of the microbial diversity in environmental or host samples, including unknown bacteria, viruses, or eukaryotic symbionts. Secondly, it provides whole genome information, which enables to detect genetic variation at a fine scale and therefore offers the potential to track the evolutionary history of the holobiont partners, including acquisition source and gain-loss dynamics of microbial diversity. One criticism on metagenomic studies investigating the genetic diversity associated with holobionts is that most of the current phylogenetic analyses using the bacterial 16S ribosomal RNA gene are led at a coarse scale. They cannot assess accurately the specificity of the association between a host and its symbionts because bacteria with similar 16S rRNA (usually above 97% sequence identity) can have substantial differences on the rest of the genome and therefore have different impacts on their host phenotypes [14]. Whole genome metagenomic sequencing allows investigating fine-scale diversity and yields robust phylogenetic information. Moreover, the whole genome information can be used to explore the phenotypic effects of symbiotic communities by using gene annotations and reconstructing holobiont metabolic networks [15].

Over the last decades, numerous computational methods have been developed to improve the analysis of metagenomic reads. These bioinformatics developments can be grouped into two main approaches: de novo genome assembly and metagenomic sequence profiling that is the grouping of sequences from one or several metagenomes into groups of the same taxonomical origin. Both of these approaches have been mainly applied to examine diversity at the species-level. If tremendous progress has been achieved in de novo metagenomics assembly [16], the inherent goal remains to build a set of consensus sequences representing the actual species in the metagenomics sample and polymorphism information is usually discarded, preventing the recovery of strain-level genomic variations [17, 18]. On the other hand, metagenomic profiling when based on reference databases is either restricted to few marker genes [19, 20] or can perform strain-level assignation only for model systems or very well studied organisms for which many strains are already characterized (for instance for biomedically important pathogens [21, 22]). Finally, reference-free metagenomic profiling approaches, also called binning approaches, are often based on previous assemblies that have already discarded polymorphism information [23, 24] or, when using co-abundance signals, may lead to incorrect binning when conserved and variable regions of a same species are sorted in different bins [25].

Overall, one of the main pitfalls of current holobiont analyses is the characterization of microbes at strain/genotype level. Apart from model communities for which comprehensive strain databases are available, fine variations in symbiont genomes are not accurately addressed by the current metagenomics-dedicated methods.

Then, a basic but efficient strategy consists in converting the problem into several non-metagenomic ones, namely analyzing each symbiont and its corresponding read subsets independently using classical genomic variation methods. The major difficulty remains to be able to partition unambiguously the read datasets, and this is definitely easier when disposing of good reference genomes for all the symbionts.

In the present paper, we present a framework designed to recover strain-level genomic variations from metagenomic reads preliminary mapped on reference genomes. When a given symbiont lacks a good reference genome, it is then built de novo from the metagenomic datasets.

To assess the potential value offered by this framework, we applied it to a biological system of moderate complexity regarding microbial communities and with good prior knowledge of the expected symbiotic diversity. The pea aphid *Acyrthosiphon pisum* is a model species for insect symbioses and shows several features which make it relevant for studying the factors structuring microbial diversity in holobionts. Pea aphids shelter an obligate bacterial symbiont, *Buchnera aphidicola* which provides the host with essential amino acids absent or scarce in the insect diet (i.e., phloem sap [26]). In addition, several secondary symbionts are commonly found in pea aphid populations at different frequencies. Some of these secondary symbionts have been shown to provide ecological advantages to their hosts, for example, by increasing protection against natural enemies or by conferring thermal tolerance [27]. While the primary symbiont is strictly maternally inherited [28], secondary symbionts are vertically transmitted with a lower fidelity and can be horizontally transmitted [29], but neither the mechanisms nor the magnitude of these events of horizontal transfers are fully understood [30]. The pea aphid actually forms a complex of at least 15 biotypes, each biotype being adapted to a specific set of host plants [31]. Estimates of divergence time between biotypes suggest that this complex may have diversified 5000–10,000 years ago, which coincides with the onset of plant domestication for agriculture [32, 33]. Population genetic analyses revealed that these biotypes form a continuum of divergence, with partially isolated host races and reproductively isolated cryptic species [32]. Several studies revealed that pea aphid biotypes also differ in their composition and frequency of secondary symbionts, but secondary symbionts seem to contribute very little to plant specialization of their hosts [31, 34–36]. In addition, strain variation has been characterized in some secondary symbionts infecting the pea aphid complex [35] and found in some cases associated with large phenotypic differences in their hosts [37, 38]. Overall, the available literature on the pea aphid symbionts indicates large variation across host populations, both in bacterial species and strains, with important functional, ecological, and evolutionary impacts on pea aphid holobionts. Although there have been recent attempts to uncover the bacterial communities associated with the pea aphid complex with deep sequencing of 16S ribosomal RNA [34, 39], no study has been yet conducted to fully characterize the diversity of pea aphid microbiota notably at different scales of organization and at a whole genome scale. The pea aphid appears to be a relevant system to develop a metagenomic framework applied to the analysis of microbial diversity and structure in holobionts. It is located at a sweet spot of complexity, with a symbiotic community of moderate size and with various modes of transmission of symbionts between hosts. It offers an interesting case of diversity partitioning between host populations through genetically and ecologically differentiated biotypes, and it is a species for which ample genomic resources are available for both the host and its associated symbionts.

In this paper, we analyzed metagenomic data from a large dataset of pea aphid-resequenced genomes to explore the extent and partitioning of microbial diversity at the different scales presented above. By mapping the reads on a set of reference genomes, we assigned the majority of the reads to microbial taxa associated with the pea aphid complex. This enabled a high-resolution inventory of the genomic diversity of bacterial symbionts found in the pea aphid complex. Variant calling and phylogenetic approaches on the whole set of symbiotic bacteria revealed contrasted levels of genomic variability and various transmission patterns between symbionts, presumably resulting from different evolutionary histories and ecologies of host-symbiont associations.

## Methods

### Biological samples

Pea aphids were collected on different plants of the Fabaceae family mainly in eastern France where host plant diversity is high but also in southern and western France (Additional file 1). Individuals were sampled as parthenogenetic (clonal) females and brought to the laboratory to initiate individual clonal lineages. After at least two generations of culture on broad bean *Vicia faba* (a plant on which all pea aphid biotypes can feed [40]), DNA was extracted from each clone in order to (i) genotype them with several polymorphic microsatellite markers, (ii) detect repeated genotypes (i.e., individuals having the same multilocus genotypes and thus presumably belonging to the same clone) and remove them from further analyses to keep a single copy per genotype, and (iii) check biotype membership of each lineage through assignment tests (see [41] for further details). Briefly, individuals with a membership equal or larger than 90% in the genetic cluster corresponding to their assigned biotype were selected for further sequencing scheme. In this study, 14 biotypes out of the 15 described for the pea aphid complex were each represented either by single or pooled individuals. Thirty-two individual resequenced genotypes encompassing 11 biotypes were those already used in [42]. This study also includes 18 new samples corresponding to pools of 14 to 35 individuals, each with a distinct multilocus genotype but belonging to the same biotype following assignment tests, representing overall 12 biotypes. Overall, the 50 samples used in this study are described in Additional file 1. Since these samples were composed of clones reared in the laboratory for at least two generations prior to DNA extraction for sequencing, their microbiota was largely composed of the heritable fraction, which was the focus of our study.

The DNA of the aphids and their microbiota was extracted using the Qiagen DNeasy Blood and Tissue Kit (Qiagen, Hilden, Germany) following the manufacturer’s instructions and sequenced in paired end using Illumina HiSeq 2000 instruments, resulting in 2× 100 bp reads with a mean insert size of 250 bp. The average read depth for the pea aphid genome was 15× for individual sequencing (42.5 million reads on average) and ranged from 20 to 50× for pool sequencing (197.5 million reads on average). FastQC files were generated for each sample, and no anomaly in the sequencing data was observed. The FastQ files of the paired reads from the 50 samples are stored and publicly available at the Sequence Read Archive of the National Center for Biotechnology Information database, under the BioProject IDs PRJNA255937, PRJNA385905, and PRJNA454786.

### Bioinformatics analyses

Full details on the analysis presented in the following parts are available on the website https://aphid-microbiome.netlify.com. This includes the source code of every custom script used during the analyses.

### Mapping-based disentanglement of holobiont genomes

Sequencing of both host and microbial DNA produces metagenomic datasets, containing reads originating from different organisms. This metagenomic context was dealt with by mapping read sets using BWA-MEM [43] with default parameters against a set of reference genomes, including the pea aphid nuclear and mitochondrial genomes, the primary symbiont genome (*Buchnera aphidicola*), and genomes of known pea aphid secondary symbionts, when available. This was the case for *Hamiltonella defensa* 5A, *Serratia symbiotica* Tucson, and *Rickettsiella viridis* and *Regiella insecticola* 5.15. For the *Rickettsia* symbiont, no closely related reference genome was available and we produced our own reference genome by de novo assembly, as explained in the paragraph below. For *Spiroplasma*, we used a draft genome previously assembled from unmapped reads of a particular pea aphid sample, as described in [42]. For *Fukatsuia symbiotica* (also named PAXS), we used the draft genome sequenced from the conifer aphid *Cinara confinis* [44, 45]*.* In addition, we included in the reference set the variant genomes of the phage APSE of *H. defensa* [46] and several plasmid sequences associated to symbionts detected in the pea aphid. In particular, we added three *Rickettsia* plasmid sequences from other insects in order to map *Rickettsia* plasmidic reads in the absence of a reference sequence for *A. pisum*. After the mapping step, several statistics were computed, including the mapping rate, the average coverage for each genome, the fraction of the reference genome covered by at least five reads, and the mean edit distance for the reads mapping on each reference genome. Reads associated to each symbiont were extracted using Samtools [47], and all downstream analyses were conducted independently and with the same settings for each symbiont. The reference genomes used for this step are summarized in Table 1. Additional statistics on the genomes used are available in Additional file 2.

**Table 1:** Summary of reference genomes used for mapping

Organism name	Sequence ID	Accession	Reference
*Acyrthosiphon pisum*	Genome	SAMN00000061	[85]
*Buchnera aphidicola*	Genome APS	BA000003.2	[86]
	Plasmid pLeu	AP001071.1	[86]
	Plasmid pTrp	AP001070.1	[86]
*Hamiltonella defensa* 5AT	Genome 5AT	CP001277.1	[87]
	Plasmid pHD5AT	CP001278.1	[87]
	Phage APSE1	AF157835.1	[88]
	Phage APSE3	EU794053.1	[46]
	Phage APSE4	EU794051.1	[46]
	Phage APSE5	EU794050.1	[46]
	Phage APSE6	EU794054.1	[46]
	Phage APSE7	EU794052.1	[46]
*Regiella insecticola* 5.15	Genome	AGCA01000000	[89]
	Plasmid pRILSR1	CM000957.1	[90]
*Serratia symbiotica* strain Tucson	Genome	GCA_000186485.2	[91]
*Spiroplasma* sp.	Genome	Upon request	[42]
*Fukatsuia symbiotica*	Genome	GCA_900128755.1	[44]
*Rickettsiella viridis*	Genome	AP018005.1	[92]
*Rickettsia* sp.	Genome	Upon request	This paper
	plasmid pREIS3	CM000771.1	
	plasmid pRF	GQ329881.1	
	plasmid pRAF	CP001613.1	
*Wolbachia* sp. *wRi*	Genome	GCA_000022285.1	[93]

### Assembly of *Rickettsia* sp. genome

Using the results of a previous mapping of pea aphid reads on the genome of *Rickettsia bellii*, we identified two samples from the *Pisum sativum* biotype with high *Rickettsia* coverage (Ps_ind1 and Ps_ind2). These two samples were pooled together, resulting in a 100× coverage on the genome of *R. bellii*. Reads that mapped on the pea aphid genome were filtered out, and the remaining ones were assembled using SPAdes version 3.11.1 [48], with default parameters. Contigs with blast matches on *Rickettsia bellii* and *Rickettsia sp* MEAM1 were extracted. To increase contiguity and genome completeness, some pairs of contigs were bridged together using the gapfiller MindTheGap [49] that performs local assembly using the whole read set.

The resulting assembly was 1,070,000 bp long (for comparison, *R. bellii* is 1.5 Mb long and *Rickettsia* sp. strain MEAM1 is 1.24 Mb), organized in 327 contigs, and had a N50 of 4483 bp. Eighty-two percent of complete genes were found using Busco v3.0.1 and the *bacteria_odb9* gene set, which is very close to the 83.7% obtained for the reference genome of *Rickettsia bellii.* Compared to *Rickettsia bellii*, we observed a major improvement of the genome coverage as 84% more reads mapped on the newly assembled genome across the whole dataset.

### Analysis and taxonomic assignation of unmapped reads

Unmapped reads were extracted using Samtools [47], and low-quality reads were removed using Trimmomatic [50] with the following parameters: LEADING:3, TRAILING:3, SLIDINGWINDOW:4:15, and MINLEN:36. Remaining unmapped reads were taxonomically assigned using Centrifuge [51]. Only assignation hits larger than 40 base pairs were kept. Results were visualized using the Pavian R package [52].

### Genome-wide variant calling

Variant calling was performed for the whole set of symbionts identified in the pea aphid samples (*B. aphidicola*, *H. defensa*, *R. insecticola*, *S. symbiotica, Rickettsia* sp., *Spiroplasma* sp., *F. symbiotica*, and *R. viridis*). It was also performed on the pea aphid mitochondrial genome, in order to capture the host matriline diversity. By essence, secondary symbionts were not present and equally abundant in all the samples, and a minimal coverage was required to run variant calling. Only symbionts with more than 10× sequencing depth and a homogeneous coverage along the genome were kept for this analysis. For instance, two symbionts in five samples were discarded because more than 90% of genomic positions were covered by less than two reads. This metric was smaller than 30% in the remaining samples.

Samtools mpileup [47] was used with options “-t DP,DPR” on the alignments to detect both SNPs and indels, and the coverage of the different alleles was reported. The generated bcf file was processed using *bcftools* [53] with options “-mv -Ov”. Abundance tables of reference and alternative alleles for each polymorphic site and for each sample were extracted for further filtering using *vcftools* [54] and processed using a custom R script (available on the https://aphid-microbiome.netlify.com). In order to remove false positive variants due to sequencing errors, rare variants were removed by applying two coverage filters: for each sample, variants covered by less than four reads or with less than 10% frequency were removed. Regions with exceptionally high or low coverage were excluded from the analysis. Genomic positions were considered of low coverage when at least 75% of samples had a coverage inferior to the median coverage of all variants along the genome. Similarly, high-coverage genomic positions were discarded when the coverage was at least five times superior to the median coverage for at least 75% of the samples. In addition, for closely related reference genomes, such as *R. insecticola*, *H. defensa*, and *F. symbiotica*, homologous genomic regions were detected by performing a pairwise blast search, and regions with a homology greater than 80% were excluded.

### Phylogenetic inference

Variant frequencies were used to compute the variant profile of each sample by selecting the most abundant allele at each site. In the case of equally covered alleles, the reference allele was kept. This situation made it difficult to determine the most abundant genotype in the sample but was rare in our dataset. We therefore decided to remove from the analysis samples in which more than 5% of variable sites yielded alleles with equal abundances. It was the case for three pool sequencing samples with low symbiotic coverage.

To investigate the evolutionary relationships between the genomes of the different samples, a phylogenomic analysis on a set of gene encoding membrane proteins was performed when an annotated reference genome was available. We first selected a list of genes, in order to compute the putative sequences for these genes in all samples. The Uniprot database was queried to retrieve DNA sequences of membrane protein transcripts (under the “Cell membrane” keyword) for the different studied symbionts (the complete list of genes used can be found in Additional file 3). Membrane proteins were selected as they are assumed to show a higher mutation rate than usual phylogenetic markers [55] and therefore are more appropriate to capture recent phylogenetic events. This query resulted in sets of 96, 118, 141, and 96 genes for *B. aphidicola*, *H. defensa*, *S. symbiotica*, and *R. insecticola*, respectively. For each sample, the putative sequences of the selected proteins were inferred by replacing the reference alleles by the alternative alleles associated to the different variant profiles.

The gene sequences of each selected protein were aligned using MAFFT [56] (v7.310, linsi mode), and the resulting multiple alignments were concatenated. The lengths of the alignments for the analyzed symbionts were 92,293 bp for *B. aphidicola*, 118,344 bp for *H. defensa*, 100,027 bp for *R. insecticola*, and 144,360 bp for *S. symbiotica*. To validate that our alignments were not subject to substitution saturation, a Xia’s test was run, as implemented in DAMBE6 [57]. Because most software of phylogenetic inference struggle to estimate branch lengths for identical sequences, we pre-processed our concatenated alignments by keeping only one sequence for each set of identical sequences. We used RaxML [58] (version 8.2.10, options -f a -# 1000 -m GTRGAMMA), a phylogenetic inference program based on maximum likelihood method, to infer the phylogeny of the samples of the considered genes. The GTRGAMMA model was used with no partitioning of the data matrix, with 1000 bootstrap iterations. Phylogenetic trees were edited and compared using functions of Ape [59] and Dendextend [60] R packages.

To cross-validate the phylogenetic relationships inferred from gene sets and also use the information contained in whole genome data, we used a clustering approach of whole genome variant profiles. Pairwise comparisons of variant profiles were performed; the numbers of differences between all pairs of profiles were then computed and divided by the total number of variants detected on the genome, as implemented in the AW-clust algorithm proposed in [61]. The distance matrix was then used to perform neighbor joining clustering and build a phylogenetic tree based on whole genome variant profile information. Tree topologies were visually compared between the gene set and whole genome approaches. For *F. symbiotica*, *Rickettsia* sp., *R. viridis*, and *Spiroplasma* sp., we did not perform a gene-based phylogeny since their reference genomes are not well assembled nor annotated. In that case, neighbor joining was performed on whole genome variant profiles to infer phylogenetic relationships between samples*.*

Outgroups were used to root the phylogenetic trees. For *B. aphidicola*, we used sequencing data of two Japanese *A. pisum* lineages, known to be highly divergent from European lineages [33]. For other symbionts, we used close-related symbiont species: *H. defensa* from the whitefly *Bemisia tabaci* (GenBank 2,777,848), *S. symbiotica* SCt-Vlc from the conifer aphid *Cinara tujafilina* (FR904230), *Spiroplasma melliferum* KC3 from *Apis mellifera* (GCA_000236085.3), *Rickettsia* sp. MEAM1 from *Bemisia tabaci* (GCA_002285905.1), and *Rickettsiella grylli* from crickets (GCA_000168295.1). For *R. insecticola*, the closest known symbiont was *F. symbiotica*, and reciprocally, the outgroup for *F. symbiotica* was *R. insecticola*.

### Phylogenetic reconciliations

We used reconciliation analyses as implemented in Jane 3 [62] to infer cospeciation and host shift events along the evolutionary history of each symbiont. The history of symbiotic relationships is commonly disclosed by comparing host mitochondrial phylogeny and symbiotic phylogeny. Many studies use phylogenetic congruence between these two types of genomes to elucidate patterns of symbiotic inheritance [63, 64]. However, achieving a high resolution in reconstructing host phylogenetic information for closely related lineages from mitochondrial DNA is challenging [28]. Since the primary endosymbiont *B. aphidicola* is known to be strictly maternally inherited [65], our strategy to overcome this limitation was to use its phylogeny as a proxy for the host mitochondrial phylogeny. *B. aphidicola* is known to have a high-mutation rate [66] as highlighted in [32] and therefore appears to be a good indicator of the recent host history [63]. In reconciliation analyses, the parasite phylogeny (in our case, the secondary symbiont) is “mapped” onto the host phylogeny (i.e., each node in the parasite tree is assigned to a node in the host phylogeny). In such a map, the diversification events of the parasites are linked to their host phylogenetic history, so that four types of events are considered: cospeciation events, host switches, sorting events, and duplication events. For the host phylogeny, we used the matriline phylogeny inferred for *B. aphidicola* gene set data which showed a better resolution than the aphid mitochondrial phylogeny, and tested for each secondary symbiont whether primary and secondary symbiont phylogenies showed significant cospeciation (indicative of vertical transmission), using gene-based phylogeny for *S. symbiotica*, *H*. *defensa*, and *R. insecticola* and neighbor joining analysis of whole genome variants for *F. symbiotica, Spiroplasma* sp., *Rickettsia* sp., and *R. viridis*. For each cospeciation analysis, we first pruned aphid samples for which the focal symbiont was detected but had insufficient read coverage to obtain reliable data for phylogenetic inferences (i.e., we did not consider the symbionts in a sample when their coverage was comprised between 1× and 10×), in order to avoid overestimating losses in the reconciliation process (i.e., considering that a symbiont was absent in an aphid sample while it was actually present but with insufficient data to perform a reliable variant calling). The focal symbiont was considered as absent when the coverage was inferior to 1×. We ultrametrized the host and symbiont trees using Grafen’s method using Ape package in R. We then ran Jane 3 [62] with the number of “generations” (iterations of the algorithm) set to 100 and the “population” (number of samples per generation) set to 100 and used default cost setting (cospeciation = 0 and all other events = 1). The cost of the best solution was compared to the distribution of the costs found in 500 randomizations in which the tip mappings were permuted at random. When the cost of the observed reconciliation is lower than expected by chance, the cospeciation signal is significant.

## Results

### Most of the microbiome diversity is captured by the mapping approach

On average, 90% of the reads were assigned by mapping to the pea aphid nuclear or mitochondrial genomes. The nuclear genome average coverage was 13× for individual sequencing and 66× for pool sequencing. 5.62% of the reads mapped on the genome of *B. aphidicola* and its plasmids, with an average coverage of 628× for individual sequencing and 3,694× for pooled sequencing. The coverages for the different secondary symbionts were very diverse and ranged from 0 (secondary symbiont was absent) to 1,300× (see Additional file 1). Presence and absence of symbionts as inferred from read depth was in agreement with the results of PCR diagnostic tests conducted for individual samples [42], and the few mismatches observed in the previous study were corrected by the choice of more appropriate reference sequences for *Rickettsia* sp., *R. viridis*, and *Spiroplasma* sp.

To further ensure that the used reference genomes were appropriate, we looked at the proportion of the genome covered by metagenomics reads and the average edit distance of reads mapping on each symbiont genome (minimum number of editing operations between the read and the corresponding part of the reference genome). Overall, more than 97% of the genomic positions of our reference genomes were covered by at least five reads. For *F. symbiotica*, we also checked that the mean edit distance of mapped reads was not larger than that of other symbionts for which we had reference genomes or we did a de novo assembly. Mean edit distance was 1.43 for *F. symbiotica* and ranged between 0.71 and 4.0 for other symbionts (average value was 1.67). Apparently, the use of a *F. symbiotica* genome assembled from another aphid host did not hamper the quality of the mapping*.*

**Fig. 1:**
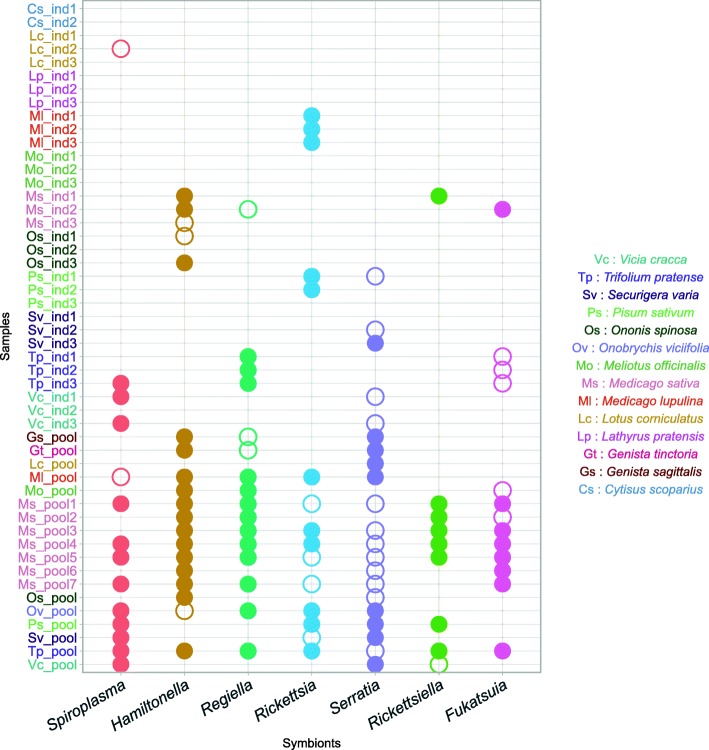
Presence/absence pattern for bacterial symbionts as detected in the metagenomic dataset. Pea aphid individuals (ind) and populations (pool) were analyzed. Empty circles indicate a coverage greater than 1×. Filled circles indicate a coverage greater than 10×, enabling phylogenetic analysis. *A. pisum* and *Buchnera aphidicola* genomes were detected in every sample

Sequencing depth data are summarized in a presence/absence matrix, as seen in Fig. 1 and are fully detailed in Additional file 1. Since only a few infected individual aphids were enough to enable the detection of a symbiont in a pooled sample, pooled data generally contained a higher richness in secondary symbionts (on average 4.28 secondary symbionts per sample for pooled samples compared to 1 for individual sequencing).

### A low number of unmapped reads validates the mapping approach

A few reads did not map onto any reference genome. The average rate of reads that did not map after quality control was 0.82% (median 0.62%, min 0.25%, max 4.76%). It confirms that mapping metagenomic reads on this set of reference genomes is able to capture most of the genomic diversity of the pea aphid complex. The unmapped rate was heterogeneous between samples and appeared linked to the symbiotic composition of the samples. Samples infected by symbionts for which a draft reference genome was used for mapping (*Spiroplasma* and *Rickettsia*) contained more unmapped reads. These reads probably originate from genomic regions absent or too divergent from these draft reference genomes. When considering samples containing only symbionts with good quality and closely related genomes, the average unmapped rate lowered to 0.69%.

The nature of those unmapped reads was further explored by conducting a taxonomic assignation of such reads with Centrifuge (version 1.0.3) [51] and its default database. Overall, only 4.9% of the unmapped reads were assigned to a taxon. The taxonomic assignation of unmapped reads is summarized in Additional file 4 and can be explored for all samples on the website https://aphid-microbiome.netlify.com/. It is in accordance with mapping results. Some reads of host or symbiotic origins that were not mapped to the appropriate reference genome were however accurately assigned by Centrifuge. Other taxa were also found by Centrifuge assignation, either because of over-assignation by the program or because some environmental organisms were sequenced along with the pea aphid and its symbionts. These reads represented a small fraction of the unmapped reads. Most unmapped reads were not taxonomically assigned by Centrifuge, probably because they contained sequencing errors or were too distant to any reference sequence in the Centrifuge database. Overall, these results indicate that the microbiota of the pea aphid complex is dominated by a few heritable symbionts and that we achieved a close to exhaustive inventory of the microbiome of our pea aphid samples.

### Different levels of intra-specific diversity for the pea aphid symbionts

The overall genomic diversity of the selected samples was estimated for each symbiont by measuring the density of variable sites between the two most different symbiont genomes in the dataset. Only pooled samples were considered in this analysis, in order to have a more comparable sample size for each symbiont.

Variant calling results are summarized in Table 2. They show strong contrasts in genomic diversity between the different symbiont taxa associated with the pea aphid complex. *H. defensa* and *R. insecticola* showed the highest diversity, with 12.6 and 16.8 variants per kilobase (kb), respectively. Conversely, genomic diversity was extremely low for *R. viridis*, with an average of 0.027 variants per kb*.* The other symbionts (*B. aphidicola*, *F. symbiotica*, *Spiroplasma* sp., *Rickettsia* sp., and *S. symbiotica*) showed intermediate levels of genomic diversity (with respectively 3.0, 1.59, 1.28, 1.19 and 1.0 variants per kb). Consequently, the lengths of the branches of the phylogenetic trees built for these various symbionts were highly variable.

**Table 2:** Summary of variant calling results. Outgroup samples were excluded to report the diversity within the dataset

Symbiont	Number of samples	Number of SNPs/kb	Number of indels/kb	Maximum distance between two samples (variants/kb)
*Serratia symbiotica*	9	1.46	0.13	1.00
*Buchnera aphidicola*	50	12.61	0.56	3.03
*Hamiltonella defensa*	16	22.16	0.91	12.61
*Regiella insecticola*	12	18.20	0.56	16.75
*Rickettsia* sp.	9	1.19	0.12	1.19
*Rickettsiella viridis*	8	0.03	0.00	0.03
*Fukatsuia symbiotica*	8	2.21	0.04	1.60
*Spiroplasma* sp.	12	1.95	0.00	1.28

### Phylogenomic analysis of *Buchnera aphidicola* from the pea aphid complex

**Fig. 2:**
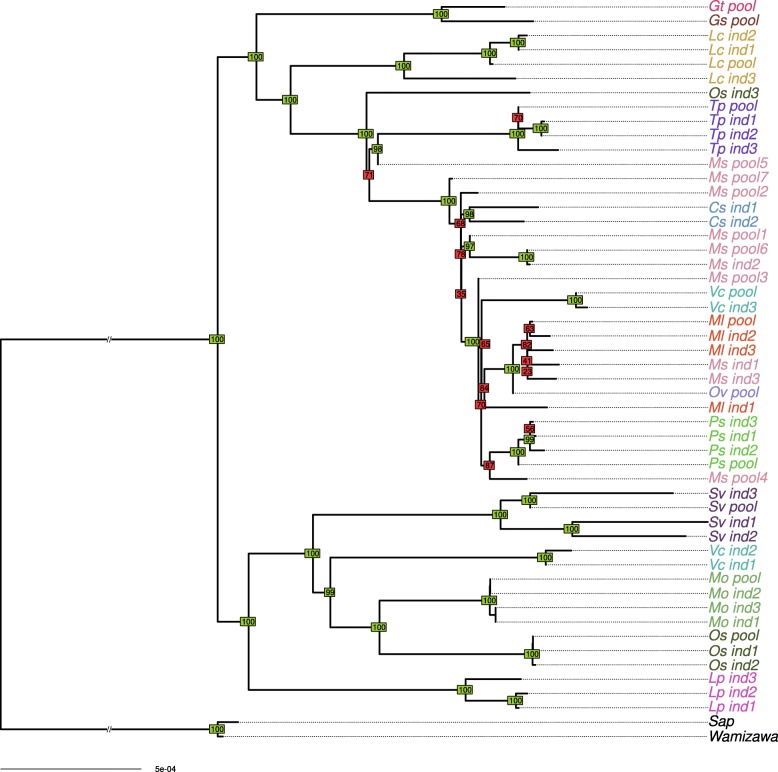
Phylogeny of *Buchnera aphidicola*. Phylogeny was inferred by maximum likelihood based on a concatenate of 96 membrane protein-coding genes. Bootstrap values above or below 90 appear in green and red, respectively

By analyzing genomic variation over the whole genome of *B. aphidicola*, we built a well-supported phylogeny of the pea aphid obligatory symbiont. No substitution saturation was detected using the Xia’s test [57] (see Additional file 5). Figure 2 shows the results of the phylogenomic analysis for *B. aphidicola* across all datasets, using maximum likelihood-based inference on a 96 gene set alignment. The tree topology obtained from the gene set was compared with a whole genome variant profile clustering. Overall, the two phylogenetic methods gave similar results, as shown in Additional file 6. The few mismatches observed between the two topologies mainly involved nodes with low support in both trees.

As previously observed using partial sequences of pseudogenes data [33], *B. aphidicola* genomes associated with the pea aphid complex are separated into two distinct clades.

Matrilines from the same biotype were generally clustered together, but some were scattered across the phylogeny (e.g., *Vicia cracca* and *Ononis spinosa* biotypes did not form single clusters). The fact that some samples from the same biotype did not cluster together likely results from incomplete lineage sorting or ongoing gene flow between biotypes [32]. When comparing *B. aphidicola* and mitochondrial phylogenies (see Additional file 7), the well-supported branches of the latter were identically retrieved on the endosymbiont phylogeny, but *B. aphidicola* phylogeny was better resolved. This confirms the suitability of using *B. aphidicola* phylogeny as a framework for examining evolutionary dynamics of secondary symbiont infections. Overall, we built a solid phylogenetic framework for *B. aphidicola* with good branch supports that we further used to contrast primary and secondary symbiont histories.

### Phylogenetic insights on the evolutionary histories of host-secondary symbiont associations

We then examined the evolutionary histories of the associations between secondary symbionts and their pea aphid hosts by comparing one by one the matriline phylogeny reconstructed from *B. aphidicola* with the phylogeny of each of the seven secondary symbionts detected with sufficient coverage in our metagenomics dataset.

**Fig. 3:**
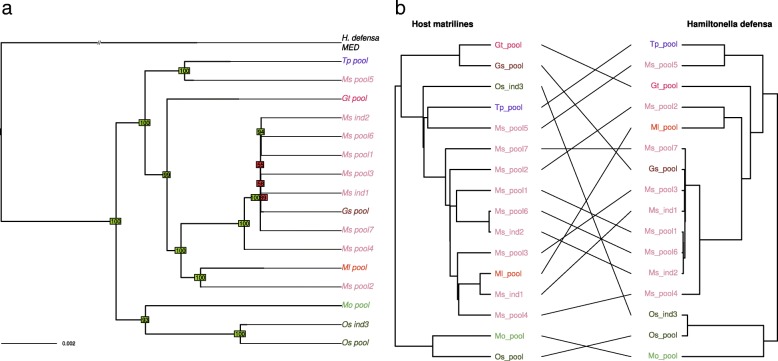
**a** Phylogeny of *Hamiltonella defensa.* Phylogeny was inferred by maximum likelihood based on a concatenate of 118 membrane protein-coding genes. Bootstrap values above or below 90 appear in green and red, respectively. **b** Tanglegram of *Hamiltonella defensa* phylogeny with host matriline phylogeny

**Fig. 4:**
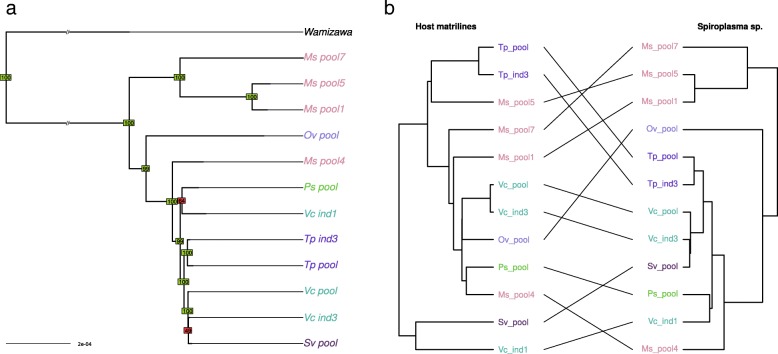
**a** Phylogeny of *Spiroplasma* sp. Phylogeny was inferred by neighbor joining based on a whole genome variant calling. Bootstrap values above or below 90 appear in green and red, respectively. **b** Tanglegram of *Spiroplasma sp.* phylogeny with host matriline phylogeny

Visual comparison of the matriline phylogeny with *H. defensa* phylogeny revealed some congruent nodes but also several differences in tree topologies indicating frequent horizontal transfers of this symbiont in the pea aphid complex (Fig. 3). Reconciliation analyses detected nine possible events of host shifts and six cospeciation events, which yielded a co-diversification scenario that is less costly than expected by chance. In addition, three events of loss were detected. This reflects mixed patterns of transmission with overall vertical transmission of this secondary symbiont along the evolutionary history of the pea aphid complex, combined with multiple events of horizontal transfers and some losses (see Additional file 8). *Spiroplasma* sp. phylogeny also showed many incongruencies with the matriline phylogeny, presumably reflecting frequent horizontal transfers (Fig. 4). Reconciliation analysis inferred eight potential host-switch events and only three cospeciation events (see Additional file 8). In that case, the cospeciation hypothesis was rejected, indicative of a shorter association of *Spiroplasma* with the pea aphid complex.

**Fig. 5:**
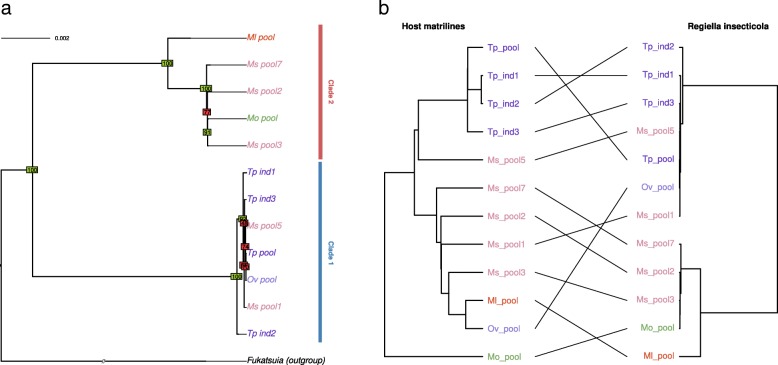
**a** Phylogeny of *Regiella insecticola.* Phylogeny was inferred by maximum likelihood based on a concatenate of 96 membrane protein-coding genes. Bootstrap values above or below 90 appear in green and red, respectively. **b** Tanglegram of *Regiella insecticola* phylogeny with host matriline phylogeny

The *R. insecticola* phylogeny retrieved two well-differentiated clades (Fig. 5). Whole genome variant calling indicated that more than 30,000 variants distinguish these two clades, while intra-clade variation was much lower, with at best 8000 variants called. These two clades may have infected the pea aphid complex separately and seem to be preferentially associated with different biotypes (*Medicago sativa* for clade 1 and *Trifolium* for clade 2). Given the low variation within each lineage relative to the large divergence between the two lineages, we can confidently assume that the acquisition of these symbionts by the different aphid hosts occurred after their divergence. The matriline phylogeny and the *R. insecticola* phylogeny showed several incongruencies within and between the two clades, suggesting frequent horizontal transfers, as suggested above for *H. defensa* and *Spiroplasma*. Accordingly, the reconciliation analysis detected 10 events of host switch and a single cospeciation event. The signal of cospeciation between *Regiella* and *Buchnera* was not significant, supporting horizontal transmission and frequent losses of events of this symbiont in the pea aphid complex (see Additional file 8).

**Fig. 6:**
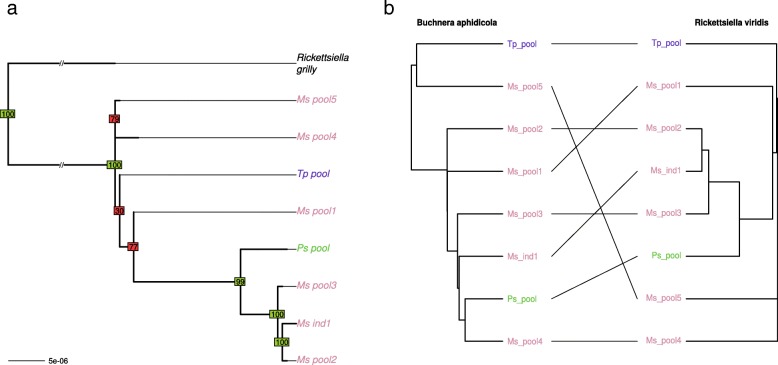
**a** Phylogeny of *Rickettsiella viridis*. Phylogeny was inferred by neighbor joining based on a whole genome variant calling. Bootstrap values above or below 90 appear in green and red, respectively. **b** Tanglegram of *Rickettsiella viridis* phylogeny with host matriline phylogeny

**Fig. 7:**
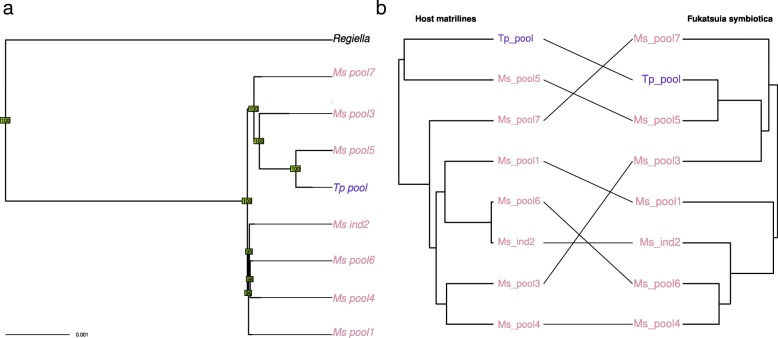
**a** Phylogeny of *Fukatsuia symbiotica.* Phylogeny was inferred by neighbor joining based on a whole genome variant calling. Bootstrap values above or below 90 appear in green and red, respectively. **b** Tanglegram of *Fukatsuia symbiotica* phylogeny with host matriline phylogeny

Despite of the low genomic diversity found for *R. viridis*, most nodes of the phylogeny are well supported (Fig. 6). Reconciliation analysis revealed only one cospeciation event along with six host-switch events. Accordingly, no significant cospeciation signal was found. This result, combined with the fact that this symbiont was found in only three biotypes of our sample and is poorly diverse, suggests a very recent history of this association in the pea aphid complex. In our sample, *F. symbiotica* was associated preferentially with the *Medicago sativa* biotype, either because of its recent acquisition, low rate of horizontal transfers, or strong incompatibilities/counter-selection in other biotypes. Phylogenetic analysis revealed a few incongruencies between tree topologies of host matrilines and *F. symbiotica* (Fig. 7). This pattern presumably reflects cases of horizontal transfer, in agreement with the reconciliation analysis that detected three host switch events. However, we found a significant signal of cospeciation (four putative events), indicative of overall vertical transmission within the *Medicago sativa* biotype.

**Fig. 8:**
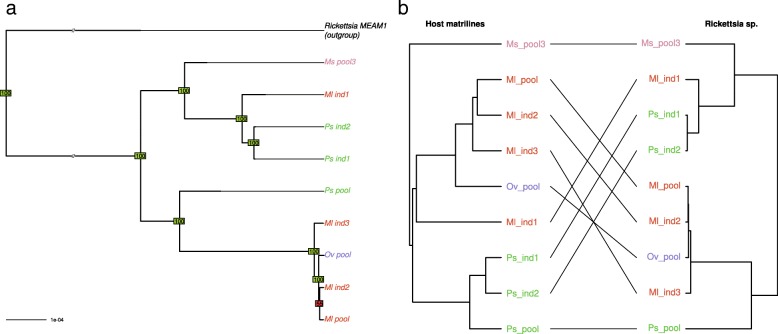
**a** Phylogeny of *Rickettsia* sp. Phylogeny was inferred by neighbor joining based on a whole genome variant calling. Bootstrap values above or below 90 appear in green and red, respectively. **b** Tanglegram of *Rickettsia sp.* phylogeny with host matriline phylogeny

Several incongruencies were observed between the phylogenies of *Rickettsia* sp. and *B. aphidicola* (Fig. 8). The reconciliation analyses uncovered four host switch events and four cospeciation events; the cospeciation signal was not significant.

**Fig. 9:**
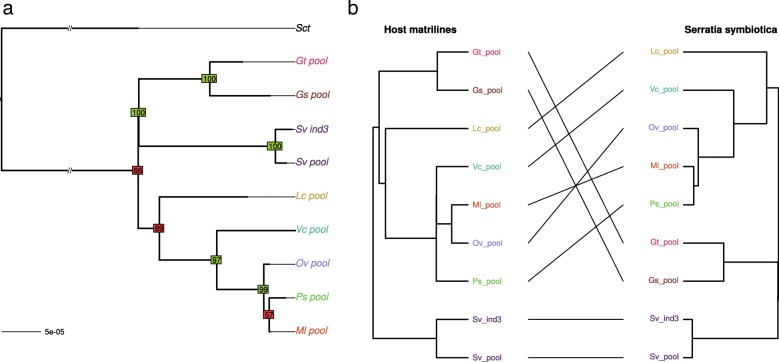
**a** Phylogeny of *Serratia symbiotica.* Phylogeny was inferred by maximum likelihood based on a concatenate of 141 membrane protein-coding genes. Bootstrap values above or below 90 appear in green and red, respectively. **b** Tanglegram of *Serratia symbiotica* phylogeny with host matriline phylogeny

The *S. symbiotica* phylogeny delineated several clades for this symbiont (Fig. 9). Nine samples were infected by this symbiont in eight different biotypes, indicating that *S. symbiotica* is represented in most of the biotypes but at a moderate prevalence across the complex. Some incongruencies were observed between the *S. symbiotica* and primary symbiont phylogenies but all involved nodes with low support on the *S. symbiotica* phylogeny. Reconciliation analyses revealed a few number of host switch events along with significant cospeciation (Additional file 8). However, the fact that *S. symbiotica* is found at a moderate prevalence suggests some failures in vertical transmission, leading to loss events in pea aphid lineages (three losses were indeed detected by the reconciliation test).

### Intra-host coexistence of two *Regiella insecticola* strains

Investigation of inter-sample phylogenetic relationships was led by considering the most abundant alleles for each sample. However, some samples may be polymorphic at some sites, with both the reference and alternative alleles covered in metagenomic dataset. While intra-sample genomic variability is expected for pooled samples, which originated each from a diverse host population, it would be more surprising for individual sequencing samples. However, we observed that genome sequences from two distinct clones of the *Trifolium* biotype (Tp_ind1 and Tp_ind2) showed a high number (32,000) of intra-sample polymorphic sites along the *R. insecticola* genome. These two samples showed no sign of polymorphism for the primary symbiont and mitochondrial genomes, excluding the hypothesis of contamination during the sequence data production.

**Fig. 10:**
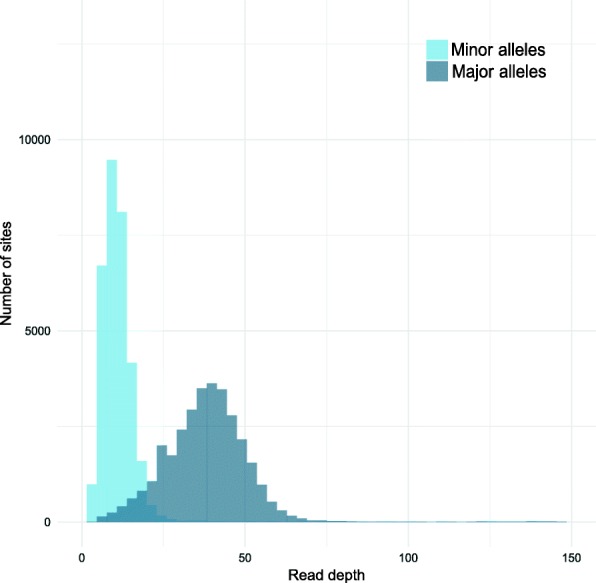
Read depth for polymorphic alleles of *R. insecticola* genome in Tp_ind1 sample. Bimodal distribution indicates the occurrence of two distinct genotypes within the same aphid host sample

Figure 10 shows the coverage distribution for major and minor alleles of *R. insecticola* in the Tp_ind1 sample. A similar distribution was obtained for the Tp_ind2 sample. These bimodal distributions suggest that two genotypes of *R. insecticola* coexist in these two samples. We estimated the read depth of the two genotypes with the most abundant genotype in Tp_ind1 covered at around 40× and the other genotype at around 10× (respectively 25× and 10× for Tp_ind2). The variant profiles for these two genotypes were close to the ones observed for the two clades of *R. insecticola* described in Fig. 5.

Sequencing data thus indicate the coexistence of two *R. insecticola* lineages inside particular samples, but it does not prove this coexistence inside individual aphids, because samples denominated as “individual sequencing” actually resulted from the sequencing of a pool of individual aphids from the same clone. Therefore, it is possible that aphids from the same clone host different symbiont genotypes. To challenge this hypothesis, we performed experimental validation on individual aphids picked in the clonal lineage maintained in culture in our laboratory. A deletion of 32 bp differentiating the two clades was identified on the contig of accession AGCA01000518 (see Additional file 9). We designed primers to amplify the region corresponding to this deletion. Electrophoresis confirmed the presence of the two haplotypes in individual aphids from the Tp_ind1 and Tp_ind2 clonal lineages, while a single haplotype was detected in aphids from the Tp_ind3 clone (Additional file 9: Figure S9). This validation confirmed the coexistence inside single individual aphid hosts of two distinct genotypes of *R. insecticola*.

## Discussion

We present here a framework to explore multi-scale genomic diversity in holobiont systems of low complexity, which is generally the case of insect holobionts. We applied this approach to metagenomic datasets of the pea aphid complex by considering microbial variation across host biotypes, among individuals of the same biotype and within individual aphids. This work allowed to extract more than 99% of the metagenomic information and to draw a complete inventory of microbes associated to the pea aphid complex, revealing a microbiota dominated by a few bacterial symbionts. Our approach also revealed for the first time a large genomic diversity among *A. pisum* symbionts, with different diversity patterns between symbiont taxa presumably reflecting distinct evolutionary histories, genomic features, transmission patterns, and ecological influences across pea aphid biotype-symbiont associations. Finally, phylogenomic analyses highlighted that frequent horizontal transfers and losses of facultative symbionts have probably been common events during the diversification of the *A. pisum* complex.

### Guidelines for analyzing multi-scale holobiont metagenomic diversity

The method proposed to finely analyze holobiont metagenomic diversity was based on the mapping of metagenomic reads on a set of reference genomes. By doing so, the entangled metagenomic read set was transformed into symbiont-specific read subsets, which enabled finer analyses such as intra-sample variability detection or strain-level diversity analyses. The method is reliable for the pea aphid holobiont, which has a restricted number of symbiotic partners, and for which reference genomes are partly available. The rate of unmapped reads was below 1% for most samples, and variations depending on the composition of symbiotic communities were observed, indicating that the availability and quality of reference genomes are important to achieve a good assignation of the metagenomic reads. When distant reference genomes are used for mapping, highly divergent regions and large insertions or deletions obviously limit the assignation success rate. Overall, mapping of metagenomics reads on a set of reference genomes (when available) or de novo assembled genomes (when coverage is sufficient), followed by a strain-level analysis of genomic variation appears to be an appropriate characterization method in the case of the pea aphid holobiont.

A large number of aphid samples were sequenced in order to investigate microbial diversity across the pea aphid complex of biotypes. However, sequencing data from host aphids did not allow accessing directly to individual bacterial genotypes, and we had to build genotypes based on the most abundant alleles in each bacterial population. In our dataset, individual sequencing samples had either a low intra-sample polymorphism or a mix of genotypes we could easily disentangle (as for example *R. insecticola* in the *Trifolium* biotype). However, pooled samples were analyzed so that only the most abundant alleles were kept to reconstruct the genotype of each symbiotic lineage. Overall, this assumption leads to underestimate the actual diversity in the pooled samples. Compared to individual host genotype sequencing, pooled sequencing allows to capture a greater diversity of symbiotic lineages, but suffers limitations in reconstructing individual bacterial genotypes, due to methodological problems in handling large intra-sample polymorphism. Despite this limitation and the fact that we applied stringent filters to discard ambiguous variants, in most cases, we could retrieve a sufficient number of reliable variants from metagenomics reads to compare symbiont diversity and to build well-resolved phylogenies.

Search for genomic variants was restricted to SNPs and short insertions and deletions. The analysis of large genomic rearrangements may bring additional information on the symbiotic genomic diversity [67]. Short variant information seems to be sufficient to reconstruct symbiotic phylogenetic trees, since most phylogenetic studies rely on gene sequences analyses, and generally do not integrate rearrangements, but this structural variation should not be neglected in order to reconstruct full genomes for the main microbial genotypes existing in pea aphid holobionts.

### Multi-scale diversity inventory of an holobiont

Previous studies on the pea aphid’s microbiota focused on the detection of symbionts using 16S rRNA PCR-based detection or 16S amplicon sequencing [34, 39]. The drawbacks of these methods are that they are restricted to bacteria, have generally low taxonomic resolution, suffer from several biases due to DNA amplification, and may be unable to identify new microbial partners [68].

To overcome these limitations, we used shotgun metagenomic sequencing, which captures whole genomic information about the host and its associated microbial community. We successfully assigned most of the reads to host and symbiont reference genomes forming the pea aphid holobiont and checked that no new bacterial symbiont was abundant in unmapped reads. Also, we found no evidence for the occurrence of *Wolbachia* in our large metagenomic dataset though this symbiont has been reported in *A. pisum* in three previous studies [34, 69, 70]. One explanation could be that none of the pea aphids used for individual or pooled resequencing projects was infected by this symbiont. Alternatively, detection of *Wolbachia* in previous studies could result from artifacts or DNA amplification from aphid endoparasitoids which may be infected by this symbiont. Because DNA was extracted from aphid clonal lineages cultured in laboratory conditions for two generations (to avoid contamination from aphid parasite microbiota and limit environmental microbes), only the inherited part of the microbiota was sequenced. In contrast with a previous study based on 16S rRNA sequencing [34], no gut associate microbe was found in our metagenomic dataset, suggesting either a low prevalence of such microbes in pea aphid populations, their loss in culture because of poor vertical transmission, or an artifact of 16S rRNA data. Finally, apart from the bacteriophage APSE, no fungal or viral associates were found. However, because of their small genome sizes, unreferenced viruses could have been missed in the unmapped-reads analysis. In addition, RNA viruses are common in arthropods and need specific detection methods [71]. Therefore, further analyses are required to in depth examination of the pea aphid virome with dedicated approaches [71]. These results altogether indicate an apparent low complexity of the pea aphid microbiota when considered at a species-level scale and are in accordance with previous works on aphids and other sap-feeder insects showing low richness of host-associated microbial communities and mostly composed of a few heritable bacterial symbionts [72, 73].

### Contrasting evolutionary dynamics of pea aphid-secondary symbiont associations

The history of the symbiosis between aphids and their primary symbiont *B. aphidicola* is well known, with a 160–280 million years old association [74]. Although *B. aphidicola* can be experimentally transferred between aphid matrilines and has been lost in a few aphid taxa [75], it is considered as a strictly maternally inherited symbiont, and no horizontal transfer has been observed so far at different phylogenetic scales [63, 76, 77]. For *A. pisum*, we observed in the present work a close congruence between mitochondrial and *B. aphidicola* phylogenies, indicating a persisting association between the host and its primary symbionts, and a codiversification of both partners in recent evolutionary time. Genome-wide analysis of *B. aphidicola* diversity in the pea aphid complex showed a diversification of pea aphid matrilines which corresponds well to the adaptive radiation that led to the complex of biotypes and confirmed previous results obtained from pseudo-gene sequences of *Buchnera* [33, 66]. Using our well-resolved *B. aphidicola* phylogeny, we were able to contrast the evolutionary trajectories of pea aphid matrilines with that of every *A. pisum* secondary symbiont and to propose different history scenarios of pea aphid-secondary symbiont associations.

Several secondary symbionts are known in *A. pisum* and other aphid species, but the nature of their association with aphid hosts is variable, from free association to co-obligatory symbiont with intermediate stages of dependency [44]. Recent data provide evidence for a higher rate of mother to offspring transmission for most of the secondary symbionts presented here [78], but some indirect proofs of horizontal transfers have also been reported [29, 35]. Their underlying mechanisms are still unclear, with host plant, natural enemies, or paternal transmission as candidate paths for horizontal transfers [30]. In this study, we showed a contrasting genomic diversity for the different symbionts, from poorly diverse symbionts such as *Rickettsiella viridis* to highly heterogeneous ones such as *Regiella insecticola*. This heterogeneity in genomic diversity could result from the combination of several factors, such as differences in evolutionary rates, population size, transmission modes, and host-symbiont association histories [78–80]. It is also very likely that these symbiotic associations are constrained by different factors including host compatibility to new infection [81] and selection [35]. For example, some symbionts like *Serratia* seem to have a wide host range [82] while others like *Fukatsuia* tend to be more restricted in terms of biotypes. In the specific case of *R. viridis*, although we cannot totally discard this hypothesis, the very low genomic variation is unlikely to result from a low-mutation rate considering the level of diversity of *R. viridis* which is two orders of magnitude less than for the other symbionts associated to pea aphids and that there is no particular mention of this pattern in the literature. Instead, this low-population genomic diversity in *R. viridis* might rather result from its relatively recent acquisition by a few *A. pisum* lineages, likely from a single of a small number of sources.

Evolutionary dynamics of symbiotic associations in the pea aphid complex were studied here by comparing phylogenetic trees of secondary symbionts with that of the obligatory symbiont *B. aphidicola*, as a proxy of pea aphid matriline phylogeny. While symbiotic species showing phylogenetic congruence with *B. aphidicola* probably reflect co-speciation with their aphid host lineages, incongruent symbiont phylogenies are expected to result from different events such as horizontal transfers or symbiont loss/gain events. Accordingly, incongruencies between matriline and secondary symbiont phylogenies were observed for all secondary symbionts considered in this study. Host switches were detected for every secondary symbiont by reconciliation analyses, supporting the hypothesis of frequent horizontal transfers proposed in previous studies on that system [35]. Reconciliation analyses also detected a few events of loss for most symbionts and those could result from failures in vertical transmission as sometimes observed in laboratory conditions [30]. With reconciliation analyses, we also found several cases of significant signals of co-speciation between secondary symbionts and their host matrilines. Since secondary symbionts of the pea aphid are maternally inherited with a generally good fidelity [78], this is not a surprising result. However, these results need to be interpreted with care as for some samples (pooling several individuals); we only reconstructed the most abundant genotype for each symbiont and might have therefore underestimated the phylogenetic diversity of the biotype-symbiont associations and the complexity of co-diversification scenarios. In any case, our approach suggests that cospeciation signals as well as the numbers of gain and loss estimated from reconciliation tests greatly differ between secondary symbionts, reflecting mixed patterns of transmission and different dynamics and durations of these symbiotic associations among the pea aphid complex. In the case of *Regiella insecticola*, we revealed an even more complex situation: *R. insecticola* populations in pea aphid biotypes encompass two highly differentiated genotypes, likely representing two distinct events of infection by symbiont strains that diverged much before the diversification of pea aphid biotypes. Horizontal transfers of these two genotypes were also detected within the pea aphid complex, indicating more recent host switch.

Overall, these evolutionary scenarios of symbiotic associations in the pea aphid complex suggest that the rate and source of horizontal transfers are very variable across symbionts, in accordance with previous studies at lower resolutions [29]. Yet, these results may be extended by larger phylogenetic studies in the pea aphid complex but also in other aphid and arthropod taxa, and by investigations of the amount and mechanisms of gain (horizontal transfers) and loss of secondary symbionts in natural populations of pea aphids.

### Intra-host coexistence of different *Regiella insecticola* strains

Our metagenomic approach on the pea aphid microbiota also revealed an unexpected level of diversity. Indeed, this study showed evidence for the coexistence of two divergent *R. insecticola* genotypes within the same individual aphid. While the within-host coexistence of symbiotic strains from the same lineage has already been reported in some arthropods [83], it has been rarely found in aphids (but see [44]). This bi-infection of *R. insecticola* strains inside individual aphids has been observed for two clones, where the two existing strains were both very different and equally abundant, facilitating detection and characterization of their infection status. However, some less obvious cases of multi-infection in other samples or by other symbionts might have been undetected. The development of dedicated techniques to analyze intra-sample polymorphism may help to better understand these events of coinfection and their evolutionary implications. The discovery of this symbiotic coinfection raises new questions concerning the effects of these strains, individually or in conjunction, on host fitness and phenotype, their localization and interaction in the aphid host, and the stability of this coinfection.

An important aspect which requires dedicated studies is how this genomic diversity in pea aphid microbiota translates into functional differences and influences the holobiont phenotype. It is known that strain-level genomic variation can have considerable consequences on the expression of the host extended phenotype. For instance, previous works demonstrated that the level of natural enemy protection provided by *H. defensa* is highly different between two *Genista* biotypes infected by genetically distinct strains of the protective symbiont [37]. Here, the reconstructed *H. defensa* phylogeny confirmed that these two *Genista* biotypes host highly different symbiotic populations, while sharing close matriline history. Genome-wide variant discovery may help to infer metabolic differences between *H. defensa* genotypes and their associated APSE phages that could cause the variation in protection levels of the hosts [84]. Similarly, a functional annotation of the genomic differences between the two highly divergent genotypes of *R. insecticola* found singly or in co-infection within the same host, may reveal different impacts on the host phenotype.

## Conclusions

We conducted a multi-scale analysis of genomic diversity associated with the pea aphid microbiota, ranging from the common species- and biotype-levels analysis, to a more innovative intra-specific analysis, and we were able to uncover the genomic diversity at each considered scale.

Improved understanding of host-microbiota relationships may benefit from large holobiont sequencing projects, and we believe the framework we developed here is applicable to other holobiont systems of low complexity. By analyzing whole genome variation in the pea aphid holobiont, we confirmed that its microbiome diversity is limited to a few inherited symbionts, but we revealed a generally large genomic diversity observed at different levels of the holobiont organization. This genomic diversity in populations of secondary symbionts seems to be mainly shaped by the dynamics of symbiotic associations, which could take multiple routes and lead to different evolutionary trajectories.

This work paves the way for new studies relying on metabolic and functional approaches and aiming to examine how genomic variation in microbiota affects host fitness and phenotypic traits. Moreover, a full understanding of the evolutionary history and ecology of symbiotic associations requires a larger investigation of the sources of genomic diversity at different geographical, temporal, and trophic scales.

Although the metagenomic framework we developed here for the pea aphid system yielded significant knowledge improvements in patterns of genomic diversity and evolution in host-symbiont associations, we pinpointed some limitations in our approach such as the availability of reference genomes and the difficulty to handle metagenomic data of high complexity. Methods to analyze fine-scale diversity from metagenomic dataset are still rare and require either well annotated reference genomes or simple communities where organisms are easy to disentangle. More advanced methods have to be developed to assess metagenomic diversity in either complex or non-model holobionts.

## Additional files

Additional file 1: Read depth of reference genomes for each sample. (XLSX 24 kb)

Additional file 2: Statistics of the symbiont reference genomes used for mapping and phylogenetic analyses. (DOCX 10 kb)

Additional file 3: Sets of membrane protein genes selected for phylogenetic inference. (XLSX 22 kb)

Additional file 4: Summary of unmapped reads taxonomic assignation by Centrifuge. (PNG 196 kb)

Additional file 5: Results of Xia’s substitution saturation test using DAMBE. (DOCX 9 kb)

Additional file 6: Comparison of symbiont phylogenies inferred by gene set phylogeny and whole genome clustering. (PDF 307 kb)

Additional file 7 : Comparison of *Buchnera aphidicola* and mitochondrial phylogenies. (PDF 225 kb)

Additional file 8: Results of phylogenetic reconciliation by Jane. (DOCX 10 kb)

Additional file 9: Intra-host detection of distinct genotypes of *R. insecticola.* (DOCX 77 kb)
