# Supplementary material for: Multi-scale characterization of symbiont diversity in the pea aphid complex through metagenomic approaches
Source: Microbiome. 2018 Oct 10;6:181. doi: 10.1186/s40168-018-0562-9 (PMC6180509; doi:10.1186/s40168-018-0562-9)
Supplement: Supplementary file 2 — Statistics of the symbiont reference genomes used for mapping and phylogenetic analyses. (DOCX 10 kb) [file 40168_2018_562_MOESM2_ESM.docx]

**Table S2 : Statistics of the symbiont reference genomes used for mapping and variant calling.** Genome coverage represents the proportion of genomic positions covered by at least 5 reads in the whole dataset.

| **Genome** | **Genome length** | **Number of contigs** | **N50** | **BUSCO completeness** | **Genome coverage** |
| --- | --- | --- | --- | --- | --- |
| *Buchnera aphidicola* APS | 640681 | 1 | 640681 | C:81.1%,F:1.4%,M:17.5% | 100 % |
| *Hamiltonella defensa* 5AT | 2110331 | 1 | 2110331 | C:98.0%,F:0.0%,M:2.0% | 99.98 % |
| *Regiella insecticola* 5.15 | 2013072 | 562 | 8294 | C :65.5%,F:8.8%,M:25.7% | 97.29 % |
| *Serratia symbiotica* | 2789218 | 388 | 201972 | C:91.9%,F:1.4%,M:6.7% | 99.80 % |
| *Rickettsia sp.* | 1054282 | 327 | 4483 | C:83.1%,F:1.4%,M:15.5% | 100 % |
| *Spiroplasma sp.* | 780201 | 160 | 6197 | C:26.4%,F:22.3%,M:51.3% | 100 % |
| *Rickettsiella viridis* | 1579736 | 1 | 1579736 | C:95.3%,F:0.0%,M:4.7% | 100 % |
| *Fukatsuia symbiotica* | 2026595 | 77 | 46872 | C:96.6%,F:0.7%,M:2.7% | 100 % |
