# Supplementary figures and images for: Multi-scale characterization of symbiont diversity in the pea aphid complex through metagenomic approaches
Source: Microbiome. 2018 Oct 10;6:181. doi: 10.1186/s40168-018-0562-9 (PMC6180509; doi:10.1186/s40168-018-0562-9)

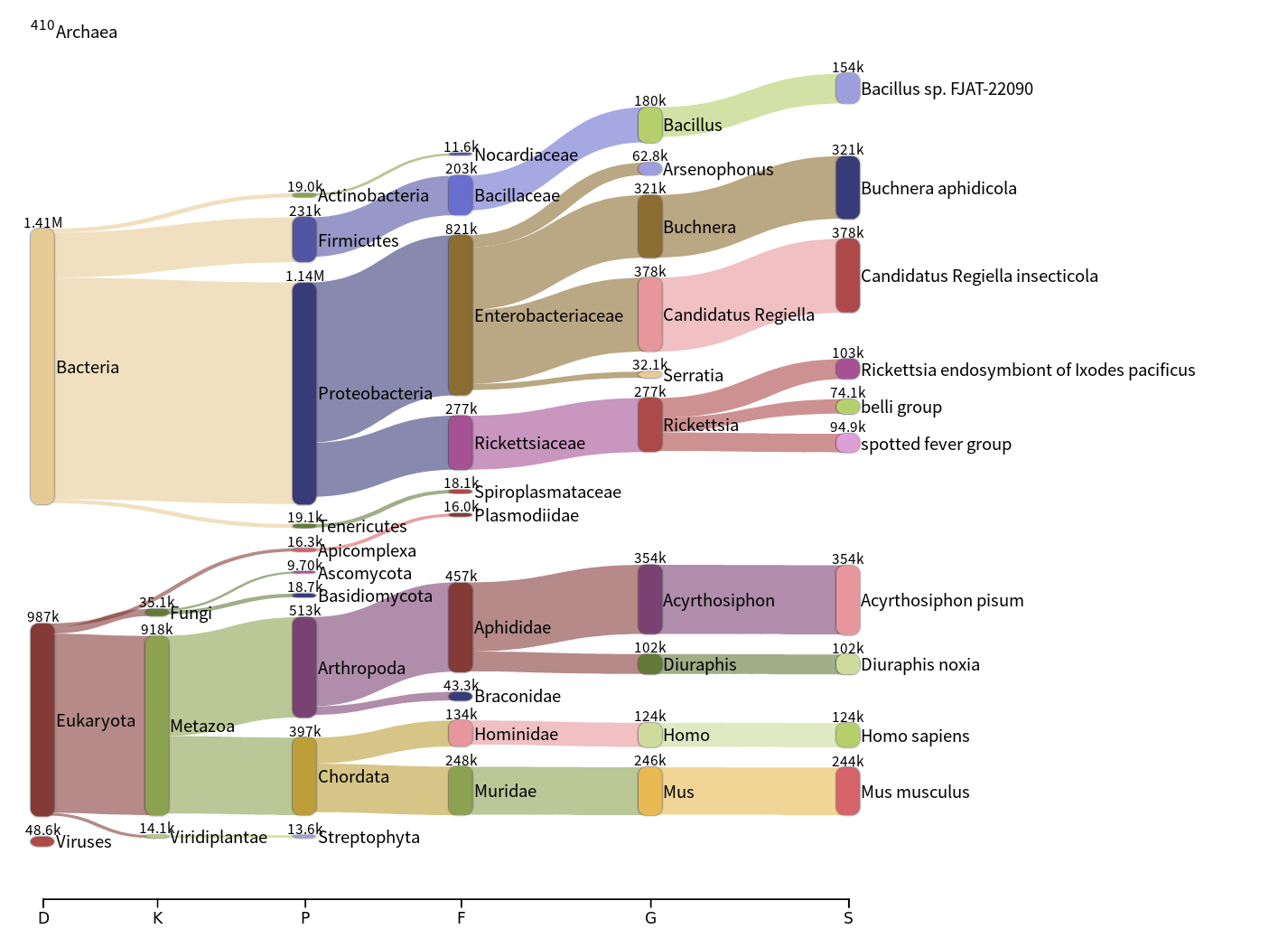

Supplement: Supplementary file 4 — Summary of unmapped reads taxonomic assignation by Centrifuge. (PNG 196 kb) [file 40168_2018_562_MOESM4_ESM.png]

a.

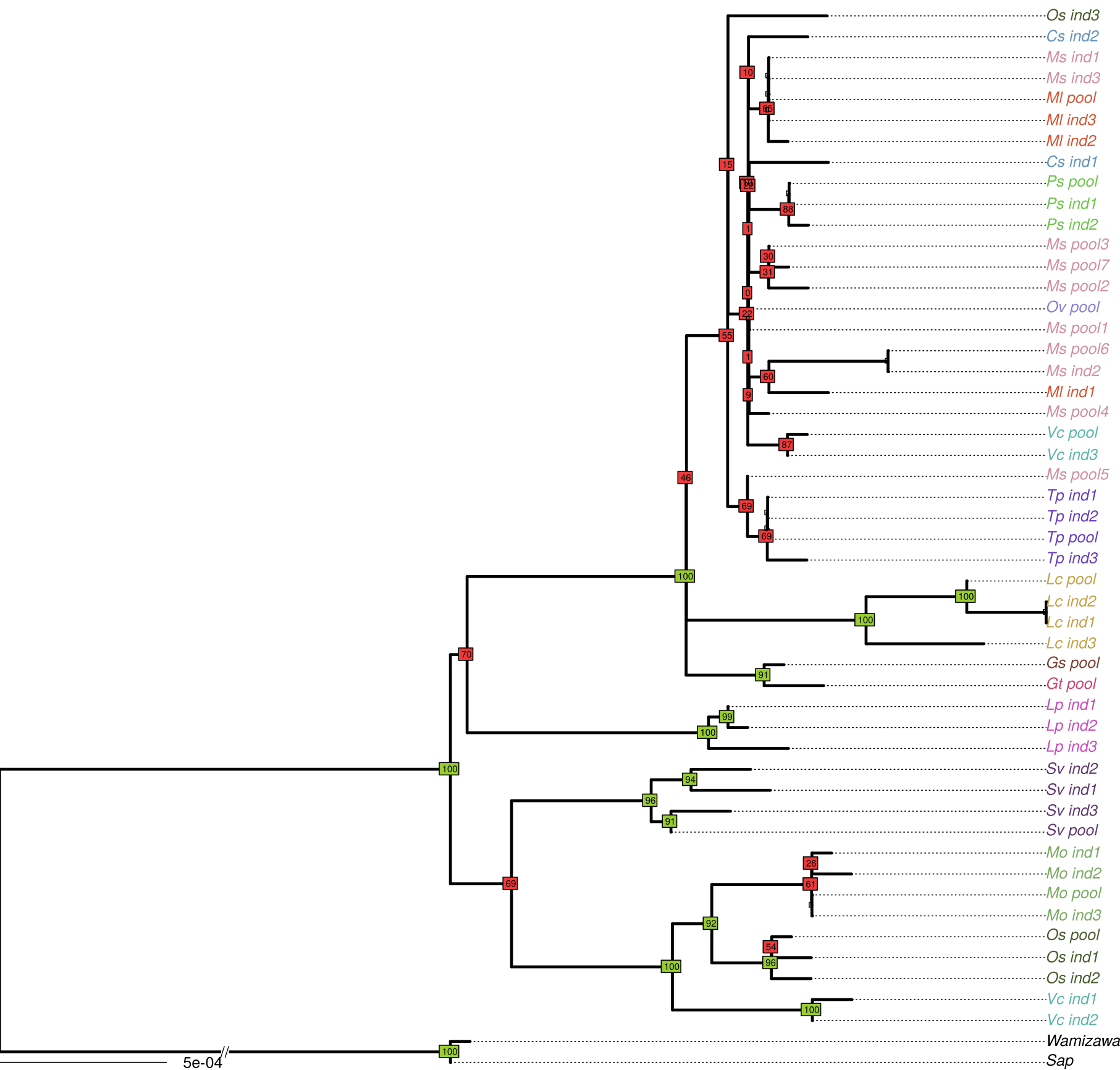

b.

A. pisum mitochondrion

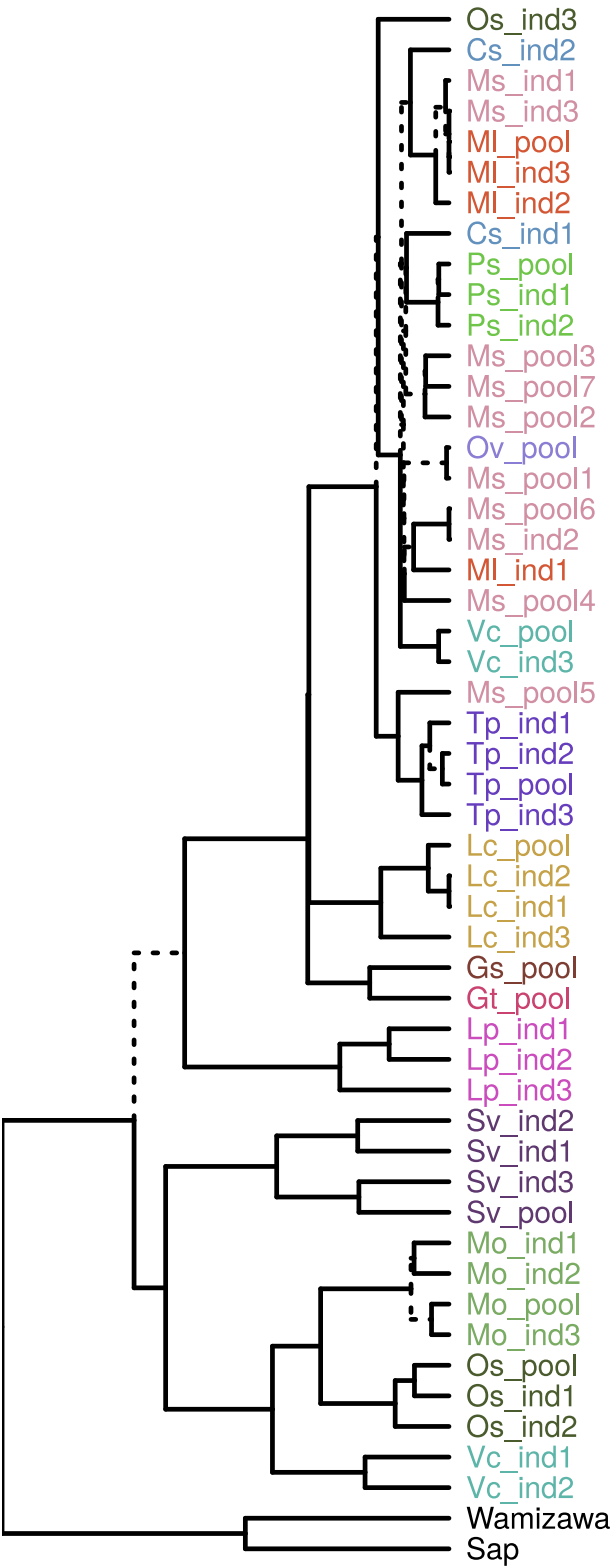

Buchnera aphidicola

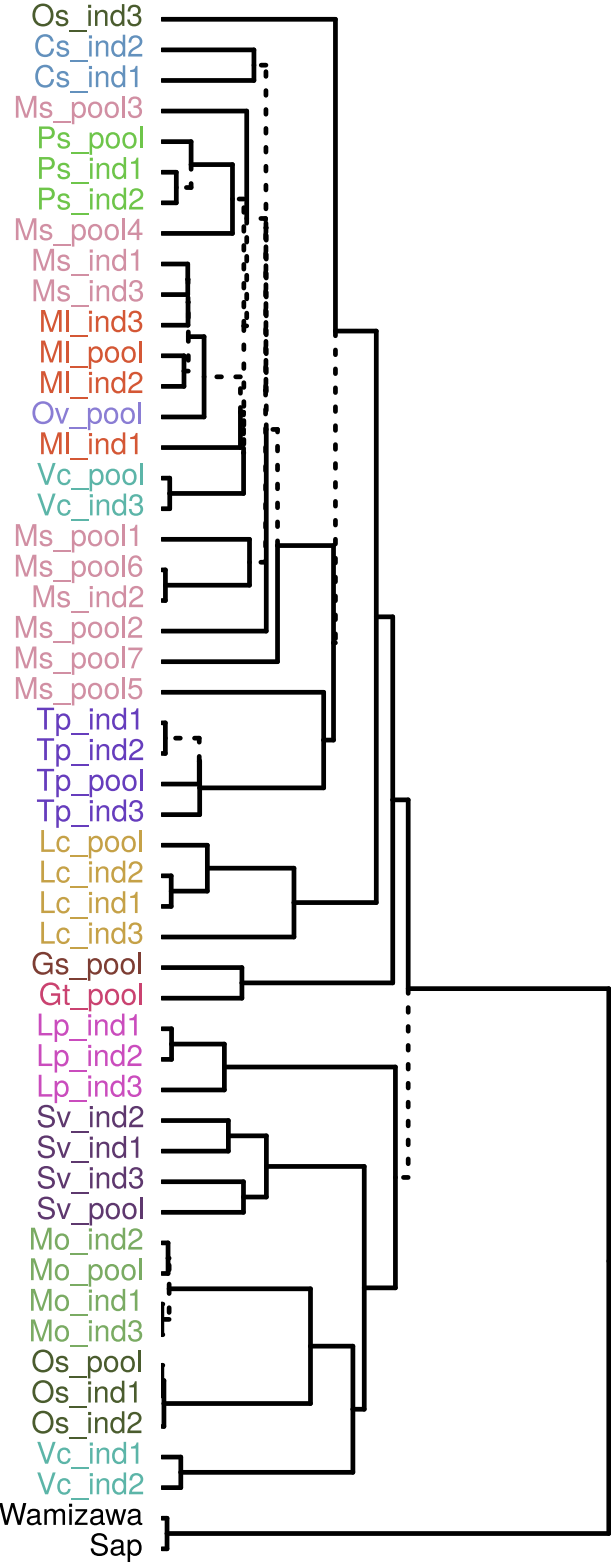

Supplement: Supplementary file 7 — : Comparison of Buchnera aphidicola and mitochondrial phylogenies. (PDF 225 kb) [file 40168_2018_562_MOESM7_ESM.pdf]
