# Supplementary material for: Multi-scale characterization of symbiont diversity in the pea aphid complex through metagenomic approaches
Source: Microbiome. 2018 Oct 10;6:181. doi: 10.1186/s40168-018-0562-9 (PMC6180509; doi:10.1186/s40168-018-0562-9)
Supplement: Supplementary file 5 — Results of Xia’s substitution saturation test using DAMBE. (DOCX 9 kb) [file 40168_2018_562_MOESM5_ESM.docx]

**Table S6** : Results of the test of substitution saturation performed by DAMBE

| **Symbiont** | **Iss** | **Iss.c** | **Two tailed P-value** |
| --- | --- | --- | --- |
| Buchnera aphidicola | 0,015 | 0,8191 | 0 |
| Hamiltonella defensa | 0,1093 | 0,8356 | 0 |
| Regiella insecticola | 0,0311 | 0,8419 | 0 |
| Serratia symbiotica | 0,0059 | 0,8454 | 0 |
