# Supplementary material for: Multi-scale characterization of symbiont diversity in the pea aphid complex through metagenomic approaches
Source: Microbiome. 2018 Oct 10;6:181. doi: 10.1186/s40168-018-0562-9 (PMC6180509; doi:10.1186/s40168-018-0562-9)
Supplement: Supplementary file 6 — Comparison of symbiont phylogenies inferred by gene set phylogeny and whole genome clustering. (PDF 307 kb) [file 40168_2018_562_MOESM6_ESM.pdf]

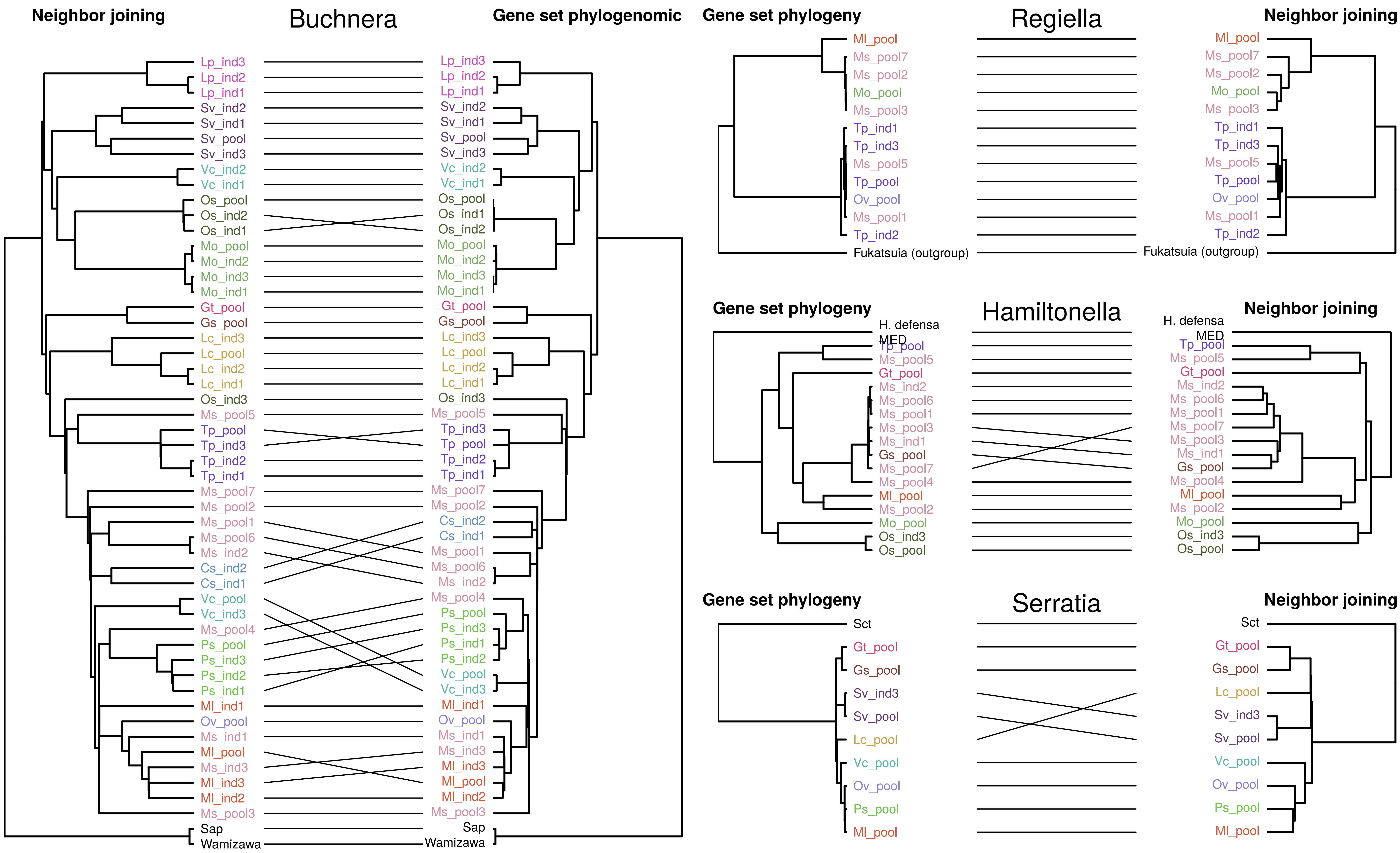
Neighbor joining
Buchnera
Gene set phylogenomic
Lp_ind3
Lp_ind2
Lp_ind1
Sv_ind2
Sv_ind1
Sv_pool
Sv_ind3
Vc_ind2
Vc_ind1
Os_pool
Os_ind2
Os_ind1
Mo_pool
Mo_ind2
Mo_ind3
Mo_ind1
Gt_pool
Gs_pool
Lc_ind3
Lc_pool
Lc_ind2
Lc_ind1
Os_ind3
Ms_pool5
Tp_pool
Tp_ind3
Tp_ind2
Tp_ind1
Ms_pool7
Ms_pool2
Ms_pool1
Ms_pool6
Ms_ind2
Cs_ind2
Cs_ind1
Vc_pool
Vc_ind3
Ms_pool4
Ps_pool
Ps_ind3
Ps_ind2
Ps_ind1
Ml_ind1
Ov_pool
Ms_ind1
Ml_pool
Ms_ind3
Ml_ind3
Ml_ind2
Ms_pool3
Sap
Wamizawa
Lp_ind3
Lp_ind2
Lp_ind1
Sv_ind2
Sv_ind1
Sv_pool
Sv_ind3
Vc_ind2
Vc_ind1
Os_pool
Os_ind1
Os_ind2
Mo_pool
Mo_ind2
Mo_ind3
Mo_ind1
Gt_pool
Gs_pool
Lc_ind3
Lc_pool
Lc_ind2
Lc_ind1
Os_ind3
Ms_pool5
Tp_ind3
Tp_pool
Tp_ind2
Tp_ind1
Ms_pool7
Ms_pool2
Cs_ind2
Cs_ind1
Ms_pool1
Ms_pool6
Ms_ind2
Ms_pool4
Ps_pool
Ps_ind3
Ps_ind1
Ps_ind2
Vc_pool
Vc_ind3
Ml_ind1
Ov_pool
Ms_ind1
Ms_ind3
Ml_ind3
Ml_pool
Ml_ind2
Ms_pool3
Sap
Wamizawa
Gene set phylogeny
Regiella
Neighbor joining
Ml_pool
Ms_pool7
Ms_pool2
Mo_pool
Ms_pool3
Tp_ind1
Tp_ind3
Ms_pool5
Tp_pool
Ov_pool
Ms_pool1
Tp_ind2
Fukatsuia (outgroup)
Ml_pool
Ms_pool7
Ms_pool2
Mo_pool
Ms_pool3
Tp_ind1
Tp_ind3
Ms_pool5
Tp_pool
Ov_pool
Ms_pool1
Tp_ind2
Fukatsuia (outgroup)
Gene set phylogeny
Hamiltonella
Neighbor joining
H. defensa
MED
Tp_pool
Ms_pool5
Gt_pool
Ms_ind2
Ms_pool6
Ms_pool1
Ms_pool3
Ms_ind1
Gs_pool
Ms_pool7
Ms_pool4
Ml_pool
Ms_pool2
Mo_pool
Os_ind3
Os_pool
H. defensa
MED
Tp_pool
Ms_pool5
Gt_pool
Ms_ind2
Ms_pool6
Ms_pool1
Ms_pool7
Ms_pool3
Ms_ind1
Gs_pool
Ms_pool4
Ml_pool
Ms_pool2
Mo_pool
Os_ind3
Os_pool
Gene set phylogeny
Serratia
Neighbor joining
Sct
Gt_pool
Gs_pool
Sv_ind3
Sv_pool
Lc_pool
Vc_pool
Ov_pool
Ps_pool
Ml_pool
Sct
Gt_pool
Gs_pool
Lc_pool
Sv_ind3
Sv_pool
Vc_pool
Ov_pool
Ps_pool
Ml_pool
