# Supplementary material for: Multi-scale characterization of symbiont diversity in the pea aphid complex through metagenomic approaches
Source: Microbiome. 2018 Oct 10;6:181. doi: 10.1186/s40168-018-0562-9 (PMC6180509; doi:10.1186/s40168-018-0562-9)
Supplement: Supplementary file 8 — Results of phylogenetic reconciliation by Jane. (DOCX 10 kb) [file 40168_2018_562_MOESM8_ESM.docx]

**Table S8: Results of the reconciliation analyses using Jane.** Secondary symbiont phylogenies are mapped on Buchnera aphidicola phylogeny. Samples whose coverage is sufficient for detection but insufficient coverage for reliable phylogenetic placement (between 1 and 10X) were pruned.

| **Secondary symbiont** | **Type of event** | | | **Cost** | | **p-value** | |
| --- | --- | --- | --- | --- | --- | --- | --- |
|  | **Cospeciation** | **Duplication & Host-switch** | **Loss** | |  | |  |
| Serratia | 4 | 4 | 3 | | 11 | | 0.02 |
| Hamiltonella | 6 | 9 | 3 | | 21 | | 0.0001 |
| Regiella | 1 | 10 | 0 | | 20 | | 0.95 |
| Rickettsia | 4 | 4 | 3 | | 10 | | 0.075 |
| Fukatsuia | 4 | 3 | 1 | | 7 | | 0.01 |
| Spiroplasma | 3 | 8 | 1 | | 17 | | 0.12 |
| Rickettsiella | 1 | 6 | 1 | | 13 | | 0.95 |
