# Supplementary material for: Multi-scale characterization of symbiont diversity in the pea aphid complex through metagenomic approaches
Source: Microbiome. 2018 Oct 10;6:181. doi: 10.1186/s40168-018-0562-9 (PMC6180509; doi:10.1186/s40168-018-0562-9)
Supplement: Supplementary file 9 — Intra-host detection of distinct genotypes of R. insecticola. (DOCX 77 kb) [file 40168_2018_562_MOESM9_ESM.docx]

**Supplementary material: Intra-host detection of distinct genotypes of *R. insecticola:***

DNA was extracted from individual aphids (three adults from each clone) belonging to clones Tp_ind1, Tp_ind2 and Tp_ind3, respectively and maintained in laboratory culture on *Vicia faba* under sustained parthenogenetic reproduction at 16h of light, 18°C. The 'salting out' extraction method [1] was used to isolate DNA from single aphids. PCR reactions were done in a volume of 10 microliters containing 1 microliter of DNA (about 100 ng) and 0.2 microliter of each primer. Primers (of sequences GAACTTTACTTCTGGCTGCCA and GCAAATTGAGGTTAGGGCCG) were used to amplify a variable region specific to *Regiella insecticola* and enabling the detection of the two clades of this symbiont based on 32 bp deletion. PCR amplicons were then separated by electrophoresis using a capillary sequencer ABI PRISM 3130xl (Applied Biosystems), following the instrument protocol GS POP7 DS33. Fragment sizes were automatically assigned by GeneMapper (version 3.5 and 3.7, Applera Corp.) and visually checked. The amplicon size is 346 bp for the first strain and 314 bp for the second (corresponding to the 32 bp deletion). Electrophoresis confirmed the presence of the two haplotypes in each of the three individuals from the Tp_ind1 and Tp_ind2 clones, while a single haplotype was detected in each of the three individuals from the Tp_ind3 clone (Figure S9).


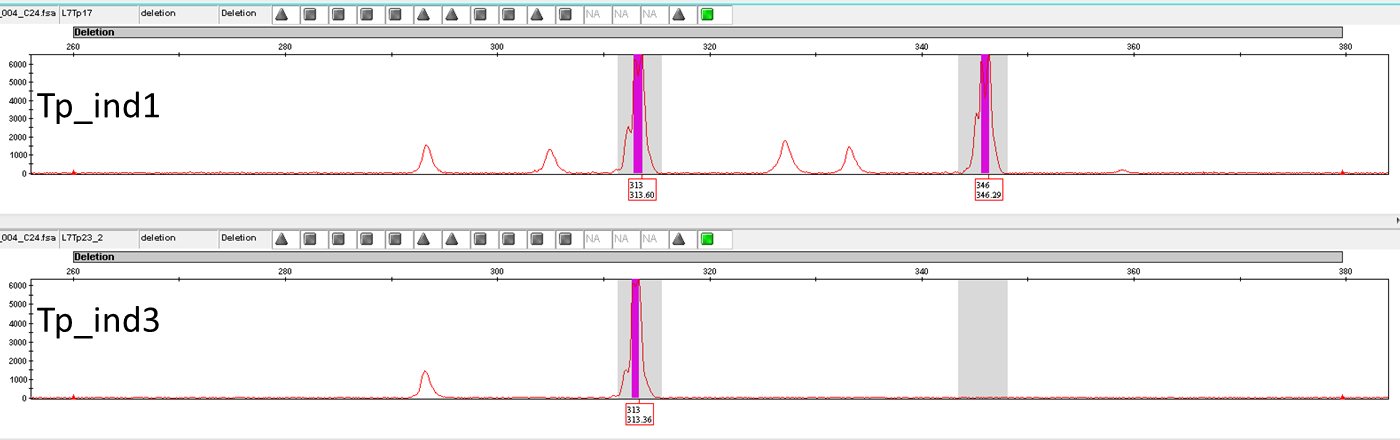


Figure S9: Chromatograms of *Regiella* *insecticola* amplicons produced by ABI PRISM 3130xl system. The purple bars on top panel peak the two fragments corresponding to the two haplotypes of *Regiella* differing by a 32bp deletion and found in Tp_ind1 clone of the pea aphid *Acyrthosiphon pisum*. In the bottom panel, only one haplotype is present in individual of Tp_ind3 clone.

[1] P. Sunnucks and D. F. Hales, “Numerous transposed sequences of mitochondrial cytochrome oxidase I-II in aphids of the genus Sitobion (Hemiptera: Aphididae),” *Mol. Biol. Evol.*, vol. 13, no. 3, pp. 510–524, Mar. 1996.
